# Experimental Study on the Shear Behavior of Precast Wall Concrete Joints with/without Dowel Reinforcement

**DOI:** 10.3390/ma13071726

**Published:** 2020-04-07

**Authors:** Qing Zhi, Xinfu Xiong, Wenjie Yang, Sha Liu, Jingang Xiong

**Affiliations:** 1 Zhongheng Construction Group Co. LTD, Nanchang 330038, China; zhjsxxf@sina.com; 2 Department of Civil Engineering, Nanchang University, Nanchang 330031, China; ywjdwyyx@126.com (W.Y.); liusha_0207@163.com (S.L.); xiongjingang@ncu.edu.cn (J.X.)

**Keywords:** precast concrete joint, shear strength, dry, epoxy resin, high strength steel bars, friction

## Abstract

The precast shear wall behavior in the serviceability and ultimate limit states depends on the shear and shear-flexural behavior of the joints between the precast components or between the precast component and footing. This study presents a series of tests on the shear strength of joints, which were applied to the interface of precast shear walls. The tested parameters included the joint types, the numbers of shear keys, the existence of high strength steel bars inserted at the joints, and the levels of confining stress. The shear capacity, stiffness, and shear transfer mechanisms of these joints were investigated. It could be concluded that the epoxied and high strength reinforcing joints had consistently higher shear strength than that of dry and plain joints. For the specimens with an inclined angle at the end of the keys of less than 60 degrees, the width of the dry joint opening may be excessively large, resulting in large shear slip and the key not shearing-off under confining stress of less than 1.0 MPa. The tested results were compared with AASHTO and other design criteria. Several formulas regarding the joint shear capacities were also proposed according to the specifications and the tested results.

## 1. Introduction

### 1.1. General

Precast concrete shear walls are generally prefabricated by off-site construction. The precast components are subsequently shipped to a construction site and then connected by grouting or welding at the joints to form an assembled monolithic structure. Therefore, the mechanical behavior of the precast concrete shear walls, such as the ultimate capacity of bending and shear and the deformational behavior, depends on the shear transfer capacity of the joints between the precast components. In addition, a potential or initial crack, shrinkage crack, construction joint, cold joint, vertical crack of corbel at the interface, and joints in other precast components (precast concrete beams, columns, and floors) and in precast concrete segmental bridges all need to transfer shear stresses caused by an external load directly. These cracks or joints are often special formations and weak parts for the components in the concrete structures. The seismic performance of precast structures is also influenced by these, which are often weak compared with cast-in-place joints under the same conditions [1,2].

**Figure 1:**
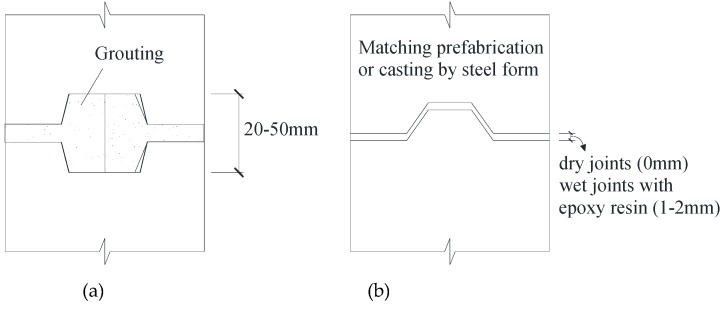
Types of joints of precast components: (**a**) Closed joints; (**b**) open joints.

The joints usually are divided into open and closed joints (as shown in Figure 1). The open joints require grouting in the reserved hole with a height of 20–50 mm. The closed joints are known as dry joints. However, in some cases, the closed joints such as shear keys without epoxy resin are also called dry joints, as defined by many researchers [3,4,5]. To improve the performance of the joints in the precast components including the shear and bond behavior, some precast design criteria also call for a rough surface or shear keys at the bonding interface between the precast component and the post-cast concrete, grouting material, or bed mortar [6,7,8]. The early application of shear keys actually started from precast concrete segmental box girder bridges. Therefore, much research has focused on the joints in precast concrete segmental bridges by studying their shear strengths compared to plain concrete and their flexural behavior [3,4,5,9,10,11]. However, few studies have been conducted on the horizontal joints of precast concrete shear walls transferring the bending moments and shear forces under horizontal wind load or earthquake action. Meanwhile, there is usually reinforcement perpendicular to the precast interface to resist the tensile force caused by the external bending moment and to provide a confining stress and dowel action, resulting in the enhancement of shear capacity and even improving the mechanical properties of the mortar [12].

With the development of precast technology, wet or dry shear keys are also applied in precast concrete structures, such as precast shear walls, to ensure the continuity and improve the overall behavior of precast structures. Furthermore, due to the rise in the technology of high strength reinforcement, the application of high strength steel bars is increasing gradually in concrete structures. However, the behavior of high strength reinforcement such as the yield plateau and ductility may be weaker than that of the ordinary reinforcement. 

**Figure 2:**
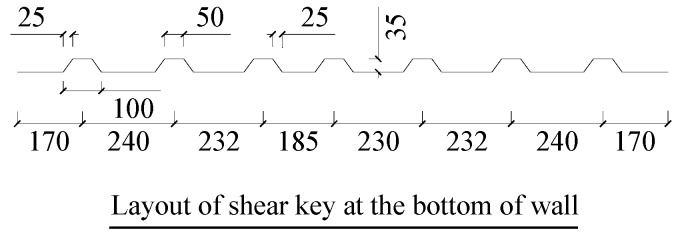

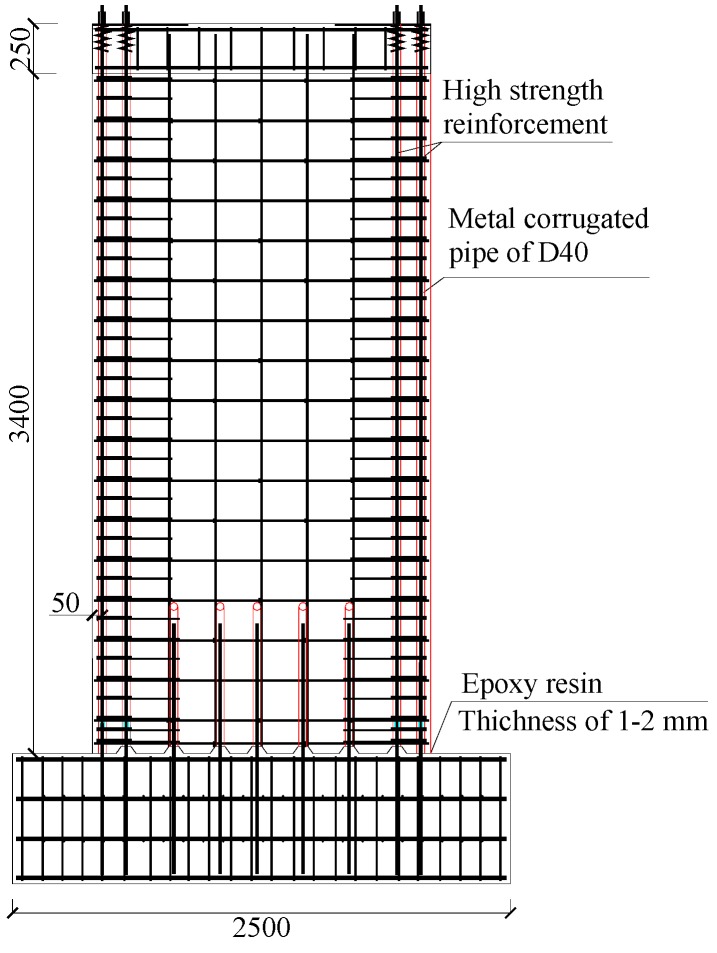
The details of the joints used in a precast concrete shear wall.

Hence, in this study, a series of tests on the shear strength of the joints with various details that were applied in precast shear walls (Figure 2) were carried out with/without high strength reinforcement across the shear plane. The tested parameters of the specimens included the joint types (dry, epoxy, cast-in-place joint, flat or shear key joint), the number of shear keys, the existence of high strength steel bars (standard yield strength of more than 600 MPa) inserted across the joint’s interface, and the levels of confining stress. Since the inserted steel bars can provide additional confining stress with the increasing crack width for the joints in addition to dowel action, they can significantly improve the mechanical performance of concrete [13] and enhance the joints’ shear strength [14]. The main layouts of the specimens were designed according to other tested specimens of shear walls. The shear capacity, stiffness, and shear transfer mechanisms of these joints were studied. Several design formulae were proposed based on the American Association of State Highway and Transportation Officials (AASHTO) and Rombach and Specker’s [15] design equations and tested results. The calculated **results** were compared with the mentioned suggested design formulae.

### 1.2. Research Significance

Previous experimental studies mainly focused on keyed joints containing dry or epoxied plain concrete joints in precast concrete segmental bridges without taking into account the effect of reinforcement across the shear plane in the joints. The joint sizes in precast concrete shear walls are different from other structural members. The work provides data that are used to illustrate the shear strength and deformational performance of precast concrete joints with different layouts and with or without reinforcement in terms of a push-off test. The tested results are compared with related design provisions. Several formulas regarding the joint shear capacities are also proposed according to the tested results and some design provisions.

## 2. Experimental Program

### 2.1. Description of Test Specimens

**Figure 3:**
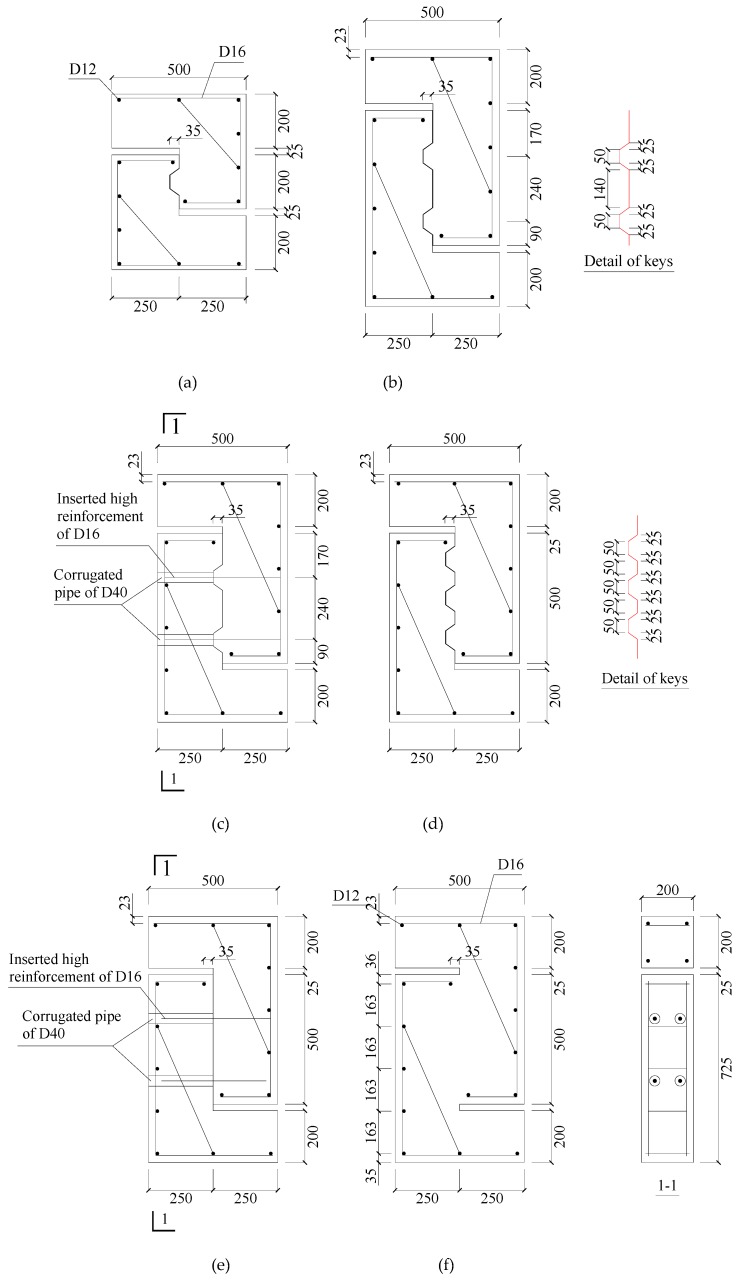
Typical dimensions of flat and keyed joints with/without reinforcement: (**a**) detail of a single keyed joint; (**b**) detail of a two-keyed joint; (**c**) two-keyed joint with reinforcement; (**d**) three-keyed joint; (**e**) detail of a flat joint with reinforcement; (**f**) monolithic cast-in-place specimen.

The joint tests were conducted by push-off without moment. Therefore, the design of the tested specimens was similar to that adopted by Zhou (2005) [3], Mattock and Hawkins (1972) [16], Issa and Abdalla (2007) [10], and Jiang (2015) [4]. The geometric dimensions of the specimens are shown in Figure 3. The effects of a flat joint, the number of shear keys, the location of shear keys, dry or epoxied condition, the dowel action of high strength reinforcement, and the prestressing level on the shear behavior were investigated. In addition, there was a monolithic cast-in-place specimen for comparison. Because the joints were adopted in precast shear walls, the thicknesses of all specimens were the same: 200 mm. However, due to the very long length in the mentioned actual shear walls, the height of tested joints was only taken as the length of the boundary element with two shear keys. However, the spacing of the keys and the inserted reinforcement in the tested joints and in the shear wall, respectively, was the same.

The test information matrix for each specimen is listed in Table 1. The identifier of the specimens is represented by four groups of characters, i.e., Mi-D (or E), -Ki (or F), -R (or N), and CIP, where M represents monotonic loading; the following letter i represents confining stress with units of MPa; D and E represent dry and epoxied joints, respectively, and the thickness of the epoxy resin is within the range of 1.4 mm–1.6 mm; K and F represent the shear key joint and flat joint, respectively, and the letter following K indicates the number of shear keys; R and N represent joints with and without high strength steel bars inserted across the tested shear plane, respectively; and CIP represents the monolithic cast-in-place specimen. For example, M1.0-E-F-R in Table 1 represents the flat epoxied joint with inserted steel bars and was tested with a confining stress of 1.0 MPa. The dimension of two-keyed specimen was simulated for the joint of the confined edge region in the precast concrete shear wall. The contacted state in Table 1 refers to the interface flatness, which was evaluated in terms of observing fractured surfaces after the test. In some cases, the specimens may not be completely contacted due to machining accuracy or construction quality, especially for the dry specimens.

The concrete material of the tested specimens was C40 with an expected compressive strength of 40 MPa after 28 days based on tests of 15 cm cube specimens according to the Standard Test Method for Mechanical Properties of Normal Concrete (GB/T 50081-2002) [17]. The actual concrete strengths ranged from 32.9 MPa to 43.5 MPa. The mix constituents of C40 concrete comprised Portland cement #425, fine and coarse aggregates, and water. The fineness modulus of sand was 2.6, and the aggregate grade was 5–16mm. The admixture of concrete was aliphatic water reducing agent. The amount of each material in each cubic meter of the concrete was 145 kg of water, 360 kg of cement, 40 kg of fly ash, 650 kg of yellow sand, 1250 kg of coarse aggregates, and 10 kg of admixture. The high strength steel bars had an actual yield tensile strength $f_{y}$ of 655 MPa, ultimate tensile strength $f_{u}$ of 833 MPa, and elastic modulus $E_{s}$ of 200 GPa based on the tension tests.

### 2.2. The Construction Process of Test Specimens

**Figure 4:**
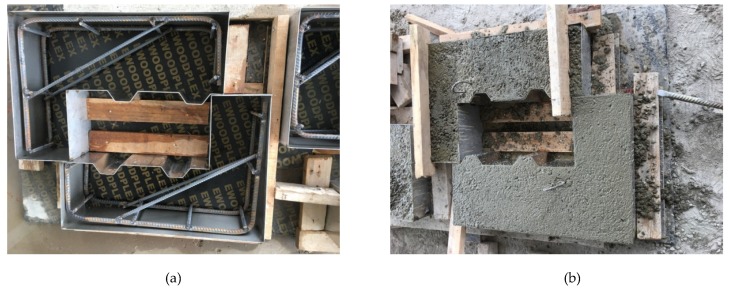
Construction process of push-off test specimens: (**a**) stainless steel machined molds; (**b**) pouring of concrete.

Since most components contained shear keys, the fabrication and installation of shear keys required high precision. Therefore, the left and right parts (female and male) of the push-off test specimens were cast with stainless steel machined molds separately or through a match-cast method (see Figure 4). 

**Figure 5:**
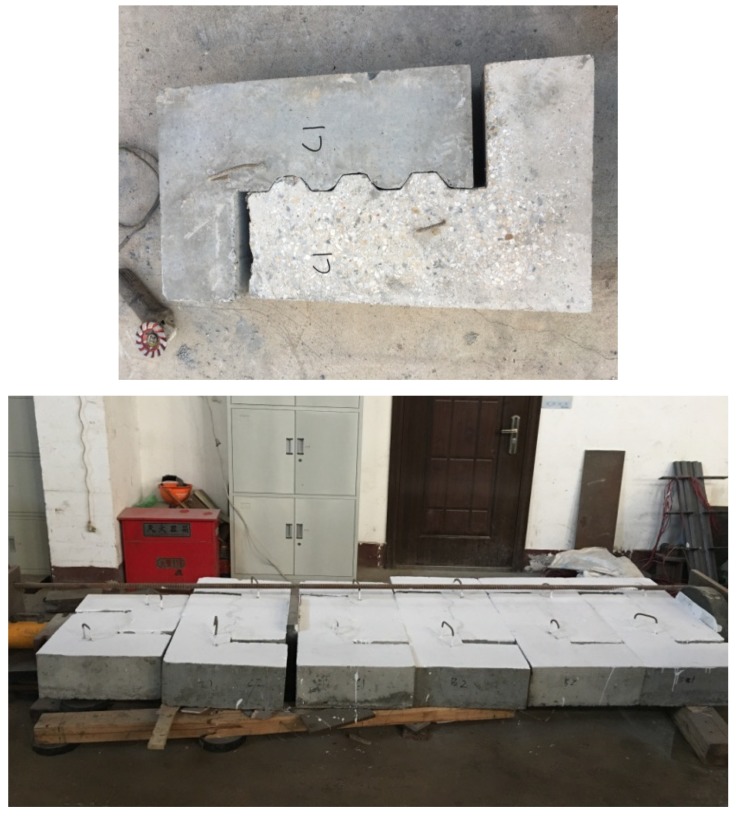
Installation process of the specimens.

After fabrication, all specimens were cured for more than 28 days. Then, the specimens were shipped to a structural laboratory for installation, curing, and testing (Figure 5). The epoxy resin used in epoxied specimens was a commercial structural adhesive from China. When smearing the epoxy resin on the joint surface, the impurities on the surface of the joint were removed by sandpaper or a polishing machine, and then, the remaining dust was wiped by medical alcohol. After volatilizing of the alcohol, epoxy resin was smeared on the surfaces. The epoxy resin was stirred evenly with the ratio of epoxy resin and curing agent being 3:1. The average magnitude of the epoxy resin thickness was 1.5 mm, which was controlled by small gaskets that were placed at the border of the surface. The gaskets could also bear the confining force before the epoxy resin was hardened. The epoxied specimens were cured in the laboratory under a confining pressure of approximately 0.3 MPa for 3 days according to the recommendations of the Post-Tensioning Institute of the United States (1978) [18] and PCI design handbook (2004) [19].

In order to investigate the effect of the dowel action of high strength steel bar on the shear behavior of joints, several joint specimens were provided with four steel bars inserted in the interface. The inserted steel bars having a diameter of 16 mm in specimens M2.0-D-K2-R and M2.0-E-K2-R were located at the middle of the shear keys. During the construction, the dowel steel bars were embedded and cast in one part of the specimen. When installing the two parts as a whole specimen, the protruded steel bars from this part were inserted into corrugated pipes in another part, which were reserved before pouring the concrete. Finally, the corrugated pipes were grouted, and then, the specimens were cured again.

### 2.3. The Setup and Test Procedure

**Figure 6:**
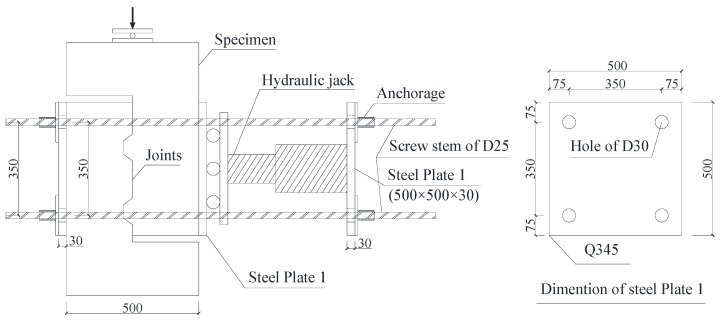
Typical setup for the push-off test.

Since the object of this study was to obtain the shear strength of precast joints in shear walls, the setup enabled the shear plane to be subjected to shear only without bending moment, which was similar to the setup adopted by Zhou [3], Mattock and Hawkins [16], Issa and Abdalla [10], and Jiang [4]. The designed and machined setup is shown in Figure 6 by taking into account the equipment, including the steel bars, anchorage device, thick steel plate with bolt holes of 25 mm diameters, roller, hydraulic jack, and test machine. Before the vertical shear force was applied to the joints of the specimens, the confining stress applied to the specimens by four steel bars and the hydraulic jack with designed values was transferred through rollers to eliminate or reduce friction force caused by the lateral force. The setup of one-keyed specimens (namely, Specimens A1–A5 as listed in Table 1) was similar to the above setup besides that a steel plate of 200 mm × 200 mm × 30 mm was used at the specimen surfaces to ensure uniform lateral confining stress. The test program for all specimens was a static test using a load control loading system that was conducted at a constant increasing load of 50 kN (for large dimensions of joints) or 20 kN (for small dimensions of joints) at the early or post-crack stage each time. Measured data during tests included the applied force by reading the stroke of the test machine, confining pressure measured by a load cell in a hydraulic pump, and the relative vertical displacement by LVDTs.

## 3. Experimental Results and Discussion

The test results of the specimens are listed in Table 2. The test results mainly included the ultimate load, average shear stress, average value of normalized shear stress, shear strength calculated according to AASHTO [6], and shear strength by Rombach and Specker’s formula. The average value of normalized shear stress was calculated by dividing the shear strength by $\sqrt{f_{c}^{\prime }}$ to consider the effect of concrete strength. 

### 3.1. Damage Process and Mode

**Figure 7:**
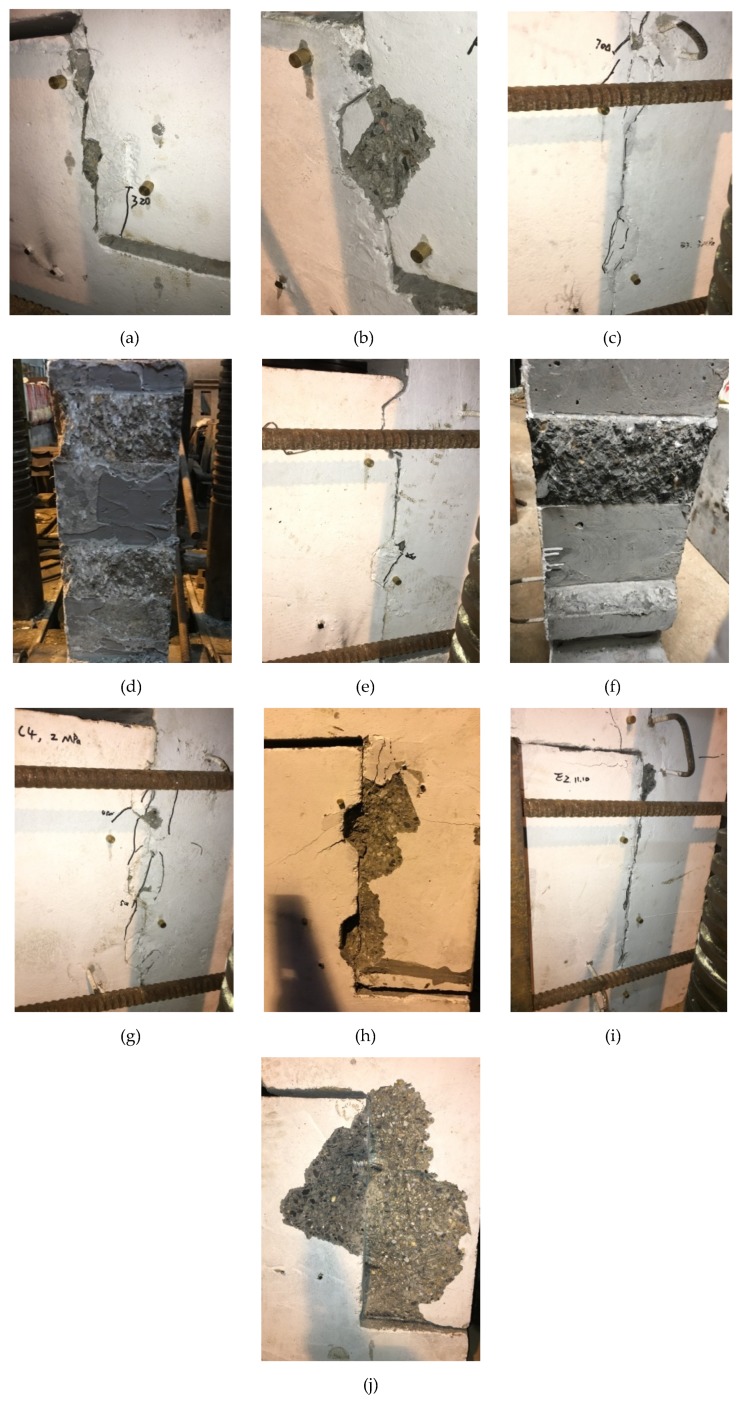
Typical crack patterns of joints during or after the tests: (**a**)M0.5-E-K1-N; (**b**)M2.0-D-K1-N; (**c**) M3.0-E-K2-N; (**d**) damaged shear plane of M3.0-E-K2-N; (**e**) M1.0-D-K2-N; (**f**) damaged shear plane of M1.0-D-K2-N; (**g**) M2.0-D-K3-N; (**h**) M2.0-D-K2-R; (**i**) M1.0-E-F-R; (**j**) M2.0-D-F-R.

Typical crack patterns of joints during or after the tests are shown in Figure 7. For the specimens in Group A with a single shear key, the surfaces of the specimens M2.0-E-K1-N and M0.5-E-K1-N were fully covered with epoxy resin. The shear strength of the epoxy resin and epoxy resin-concrete interface was larger than that of the concrete. As a result, an initial crack appeared during the loading and then developed within the concrete layer adjacent to the epoxy resin layer. It illustrated that the shear strength of epoxied-keyed joints with fully-covered epoxy resin was determined by the tensile strength of the concrete, which agreed with the test results of Zhou [3]. For the dry specimens M2.0-D-K1-N and M1.0-D-K1-N, their shear keys were sheared off at the maximum shear loads. For the specimen M0.5-D-K1-N with confining stress of 0.5 MPa, however, due to the small lateral force, the shear locking phenomenon did not occur in the shear key, resulting in the slipping of the shear plane at the interface without shearing-off failure in the shear key. Only a crack at the bottom of the shear key of approximately 60 degrees appeared before slipping failure. It could be found that the shear strength of a shear key with this configuration could not fully be developed with confining stress less than 0.5 MPa (we would also find that the confined stress of 1.0 MPa may also give rise to the slipping of the shear plane for other dry joints in subsequent tests). 

For the specimens in Group B with two shear keys and without inserted reinforcement, the failure mode of specimen M2.0-E-K2-N was similar to that of the single-keyed epoxied specimen M2.0-E-K1-N where the whole failure plane was located in the male part of the specimen. For the specimen M3.0-E-K1-N with partial contact filled by epoxy resin in the interface, two areas of approximately 130 mm × 130 mm and 100 mm × 50 mm were damaged, which led to the detaching between the epoxy and concrete, as shown in Figure 7d. This detaching may result from the thin thickness of epoxy resin under construction where the epoxy resin was usually used to fill the gap in the interface to form a uniform shear stress distribution on the interface and obtain higher shear strength than dry joints. As for dry joint M1.0-D-K2-N, since the shear stress distribution under push-off loading was not uniform due to the unavoidable gap in the interface and the small confining stress of 1.0 MPa, one of the shear keys was shearing-off, and the other was slipping with a large gap opening. Therefore, the ratio of height to width of the shear key (that is the inclined angle at the end of the key) also had an important effect on the shearing-off failure mode, especially in the case of small pressure. Hence, it was reasonable to take the inclined angle at the end of shear key as no more than 60 degrees as specified in the Technical Specification for Precast Concrete Structures of China (TSPCS) (2014) [8]. However, this failure mode in the joints of precast concrete shear walls will usually not appear due to the large axial compression ratio and large size of the section with reinforcement when adopting this kind of configuration in this study, which was also applied by Jiang (2015) [4] in precast concrete segmental bridges. From the failure modes of M2.0-D-D2-N and M3.0-D-K2-N with confining stress no less than 2.0 MPa, it could be found that all shear keys were sheared off. Larger confining stresses will change the crack patterns and the failure modes [4,5]. 

For the specimens in Group C with three shear keys, the shear strength of the dry specimen was higher than that of specimens with single and two shear keys. The failure modes of the specimen M2.0-D-K3-N was similar to the dry specimen M2.0-D-K2-N with two keys. The three shear keys were all sheared off. However, the keys did not fail simultaneously due to the construction imperfections. The shear strength of M1.0-E-K3-N was close to that of the cast-in-place specimen M1.0-CIP-N with the same confining stress. The failure mode of M1.0-CIP-N of the monolithic cast-in-place joint was relatively simple. Because there was no transverse reinforcement across the shear plane in the cast-in-place specimen, the failure was brittle fracture after reaching the ultimate load during the test accompanied by a vertical crack.

For the specimens in Group D with two shear keys and inserted reinforcement, the external shear load of the specimen M2.0-D-K2-R was resisted by the combined dowel action of reinforcement and shear keys. However, due to the existence of the unavoidable gap in the dry joint, the shear keys were not fully contacted so that the load was mainly resisted by the dowel action of steel bars at the initial loading stage. The shear keys and dowel reinforcement worked together after the two components of the specimen were fully contacted, resulting from the bending and shear deformation of dowel steel bars. The local stress of the concrete under the inserted steel bars was relatively large, leading to the spalling of concrete adjacent to the dowel reinforcement at the later loading stage. Moreover, the deformation capacity of the reinforced joints was more than twice as the above unreinforced joints, resulting in a ductile failure. The shear keys were not totally sheared off when compared with other specimens. There were two longitudinal cracks along the dowel reinforcement because of bond stresses and the inclined concrete compression. The specimen M2.0-E-K2-R with epoxy resin was damaged by local failure at the loading end (820 kN) with only several tiny vertical cracks observed, so the true ultimate shear load was not obtained.

For the specimens in Group E with flat joints, the failure modes of M2.0-D-F-R and M1.0-D-F-R were almost the same. Their concrete cover was split seriously as indicated in Figure 7j which was also similar to the specimen M2.0-D-K2-R. However, the shear strengths were 71% and 52% for M2.0-D-K2-R with shear keys, respectively. These were 60% and 44% for specimen M1.0-E-F-R with epoxy resin and inserted steel bars in Group E, respectively. The damaged shear surface was located at the concrete layer near the epoxy resin layer, similar to other epoxied specimens. 

### 3.2. Normalized Average Shear Stress and Relative Displacement

**Figure 8:**
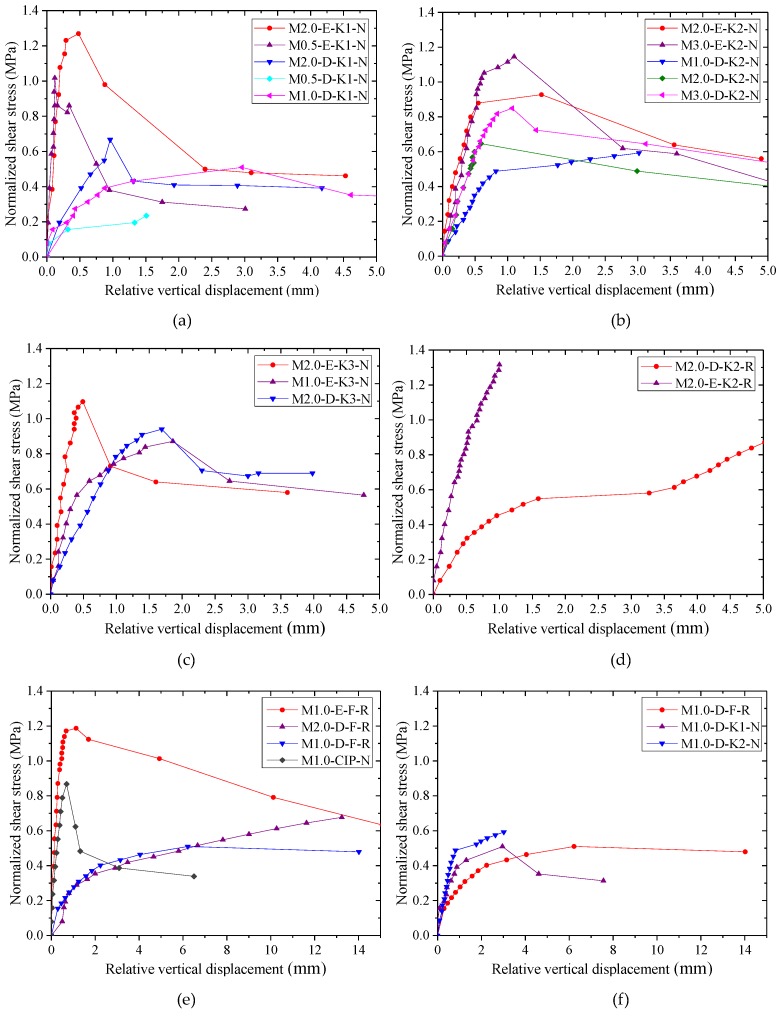
Normalized average shear stress-relative vertical displacement relationships of specimens with dry and epoxied joints: (**a**) specimens with single shear key; (**b**) specimens with two shear keys; (**c**) specimens with three shear keys; (**d**) specimens with two shear keys and with dowel reinforcement; (**e**) flat joints and integral cast-in-place specimens; (**f**) dry flat reinforced, dry single-keyed and two-keyed specimens.

**Figure 9:**
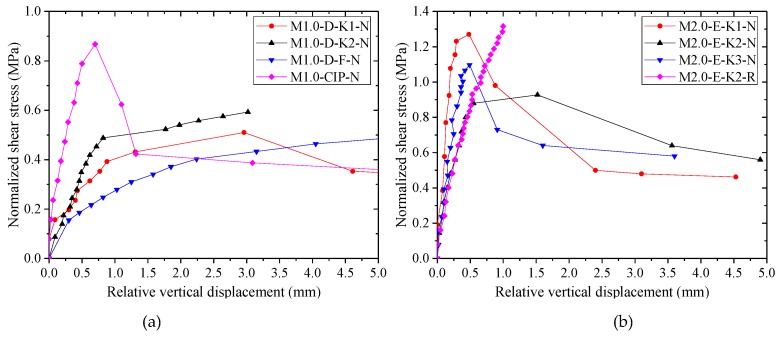
Normalized average shear stress-relative vertical displacement relationships of specimens expressed by dry and epoxied specimens, respectively: (**a**) dry and cast-in-place specimens with confining stress of 1.0 MPa; (**b**) specimens with epoxy resin and with confining stress of 2.0 MPa.

According to the recorded data of shear load-relative vertical displacement during the tests, the normalized average shear stress-relative vertical displacement relationships of all specimens are shown in Figure 8 and Figure 9. 

In Figure 8, it can be found that the shear strength and the initial stiffness of the specimens with epoxy resin were both larger than those of dry specimens whether the specimens had single, two, or three shear keys. The shear capacities of specimens were increased with the increase of confining stress. However, the effect of confining stress on the initial stiffness was not significant, especially for epoxied specimens. When the load of the unreinforced epoxied specimens exceeded the maximum load during the test, the shear capacity dropped quickly, and the failure mode was brittle failure. At the later loading stage, most of the specimens deformed with residual bearing capacities provided by friction produced by the external steel bars, which were similar to those of dry joints. In addition, for the specimens with epoxy resin, their displacement corresponding to the ultimate load was mainly concentrated around 0.5 mm, which was close to that of the cast-in-place specimen M1.0-CIP-N. However, for the dry joints, the displacement corresponding to the ultimate load ranged from approximately 1.0 mm to 3.0 mm, which was dependent on the imperfect gap opening.

Furthermore, it can be found in Figure 8d,e that the dowel reinforcement producing dowel action and confining stress could increase the shear strength by 41% and 36% for M2.0-E-K2-N (compared with M2.0-E-K2-R) and M1.0-CIP-N (compared with M1.0-E-F-R), respectively, and bring about ductile failure. The initial stiffness of reinforced joint was slightly large compared with the unreinforced joint. The loads of dry specimens M2.0-D-K2-R, M1.0-D-F-R, and M2.0-D-F-R increased till the displacement exceeded 15 mm. In this process, except for the dowel action explaining the increasing trend, the crack width of the joints became gradually large, resulting in the elongation of external and internal (inserted) steel bars, so as to increase the lateral confining stress and the shear strength. The shear strength of M1.0-D-F-R with confining stress of 1.0 MPa was similar to that of M2.0-D-F-R with confining stress of 2.0 MPa at the early loading stage. After reaching the displacement of 6.5 mm, the load of M2.0-D-F-R was larger than that of M1.0-D-F-R. During the test, it was found that the shear surface of M2.0-D-F-R was not closed completely, and there was a little gap between the two parts of the components, which was caused by the inserted steel bars being stuck in when this specimen was installed. Subsequently, the inserted bars were bent by shear force with the increasing load until the gap became small. This error in the construction process was obviously harmful for the precast concrete components because it influenced the mechanical behavior and the durability of the precast joints and may generate cracks parallel to the reinforcing bars when the joint was subjected to small loads. 

For the dry specimens M1.0-D-F-R, M1.0-D-K1-N, and M1.0-D-K2-N with identical confining stress, as shown in Figure 8f, although the ductility of M1.0-D-K1-N and M1.0-D-K2-N was smaller than that of M1.0-D-F-R with reinforcement, the initial shear stiffness of the keys was higher than that of dowel reinforcement. 

The normalized average shear stress-relative vertical displacement relationships of different specimens with the same confining stress are shown in Figure 9. The dry specimen M1.0-D-K1-N with a single key indicated lesser shear strength than specimen M1.0-D-K2-N with two keys, which did not obtain an identical result to that of Zhou (namely, the normalized average shear stress of the dry single-keyed joint was larger than that of specimens with multiple shear keys under the same condition because of the incomplete contact of shear keys), which may be due to imperfections in the fabrication and the discreteness of the tested results.

## 4. Shear Capacity of the Joints

### 4.1. Shear Strength of Dry Keyed Joints 

**Figure 10:**
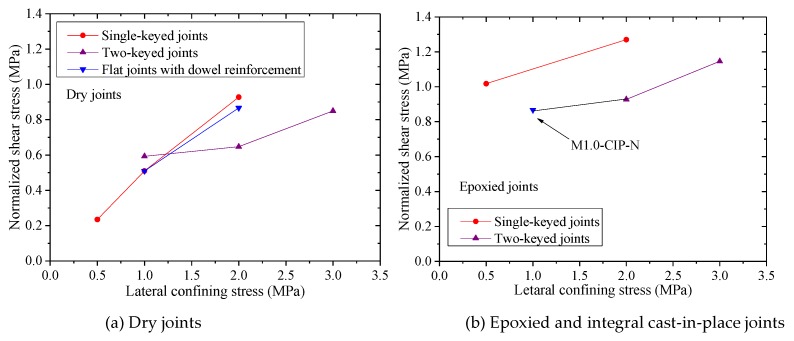
Normalized average shear stress-lateral confining stress relationships of the joints of the same type under different confining stresses: (**a**) dry joints; (**b**) epoxied and integral cast-in-place joints.

According to other researchers [4,15], the relevant specifications (AASHTO, ACI, and TSPCS of China) [6,7,8] and the shearing-off failure modes in the study, the shear capacities of the joints were increased almost linearly with the increase of lateral confining stress. The normalized average shear stress-lateral confining stress relationships of the joints with the same type under different confining stresses are shown in Figure 10. There was usually a vertical failure plane when the joint was damaged by shearing-off. The shear capacity of the dry joint was composed of the shear contribution of the contact flat parts and the shear keys. The shear strength provided by flat parts depended on the friction coefficient and confining stress. Buyukozturk (1990) [20], Gaston and Kriz (1964) [21], and Tassios and Vintzēleon (1988) [22] studied the concrete friction coefficient in the early stage and took it as 0.6. AASHTO and the ACI code also take the coefficient as 0.6. The shear capacity of keys can be obtained through subtracting the shear strength of flat parts of concrete from the total shear capacity of the joint. The test results were compared with the design formula of AASHTO as listed in Table 2. The design formula of AASHTO was mainly used to calculate the shear strength of shear keyed joints in precast concrete segmental bridges.

(1) $$\tau_{f}=\mu \sigma_{n}$$

(2) $$V_{j}=A_{k}\sqrt{f_{c}^{\prime }}\left(0.9961+0.2048\sigma_{n}\right)+0.6A_{sm}\sigma_{n}\left(MN\right)$$

where $\tau_{f\;}$ is the shear strength of a flat joint, μ is the coefficient of friction, $\sigma_{n}$ is the confining stress, $V_{j}$ is the shear capacity of a joint, $A_{k}$ is the area of the failure surface of all keys, $f_{c}^{\prime }$ is the concrete strength with units of MPa, and $A_{sm}$ is the contacting (flat) smooth area of the joint. 

The calculated formula suggested by Rombach and Specker [13] is expressed as follows:

(3) $$V_{j}=0.14f_{c}^{\prime }A_{k}+0.6A_{j}\sigma_{n}$$

where $A_{j}$ is the area of the entire joint surface, $A_{j}=A_{k}+A_{sm}$. The calculated results of the above equations are also listed in Table 2. It should be noted that the above equations were usually used for the joints with shear keys that did not include the flat joints. To study the applicability of the design formulas of AASHTO and the suggestion by Rombach and Specker, the shear strength of flat joints was also computed in this study by taking $A_{k}$ as 0 m^2^ and taking $A_{sm}$ as $A_{j}$, respectively. From the calculated results in Table 2, it can be found that the calculated results of dry joints with two and three keys were in correspondence with the recorded results, and the error was only approximately 5%. For the epoxied joints, the calculated results by the formulae were obviously small. For the dry joints with a single shear key, the measured shear strength was significantly lower than the results calculated by Equations (2) and (3), especially when the confining stress was low (that was M0.5-D-K1-N with a confining stress of 0.5 MPa in Table 2). Hence, the shear strength of this kind of single-keyed joint was not always fully developed in this case due to the slipping of the key. 

**Figure 11:**
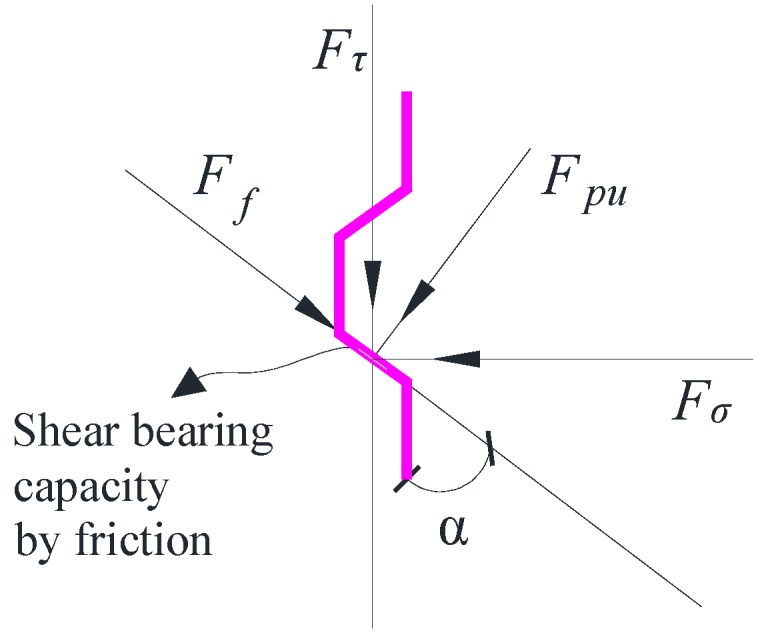
Shear bearing capacity by friction at the key interface.

**Figure 12:**
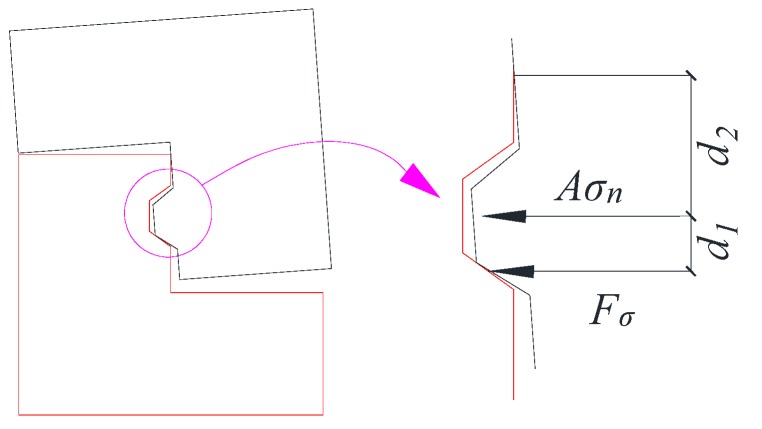
The key interface state after the slipping of the key.

From the test results, the failures of shear keys could be divided into two types: failure caused by the fracture of keys with high confining stress and failure caused by slipping with small confining stress. When the shear bearing capacity by friction was lower than the bearing capacity by the shear key, which could be calculated by the design formulae such as Equation (2), slipping would be observed. The friction at the key interface was dependent on the applied confining stress and inclined angle α, as shown in Figure 11. The shear bearing capacity by friction could be computed according to the force equilibrium equations as follows:

(4) $$F_{pu}\cos\alpha -F_{f}\sin\alpha =F_{\sigma }$$

(5) $$F_{pu}\sin\alpha +F_{f}\cos\alpha =F_{\tau }$$

where $F_{pu}$ is the force perpendicular to the contact surface of the key, $F_{f}$ is the force parallel to the contact surface, $F_{\sigma }$ is the horizontal confining force, which can be obtained based on the geometrical relationship and the equilibrium of forces in Figure 12 by taking into account the equilibrium condition of the moment, $F_{\tau }$ is the shear bearing capacity by friction at the interface, and α is the inclined angle of the key. Thus, the shear bearing capacity $F_{\tau }$ by friction can be expressed as:

(6) $$F_{\tau }=\frac{\sin\alpha +\mu \cos\alpha }{\cos\alpha -\mu \sin\alpha }F_{\sigma }$$

where μ is the friction coefficient:

(7) $$\mu =\frac{F_{f}}{F_{pu}}$$

**Figure 13:**
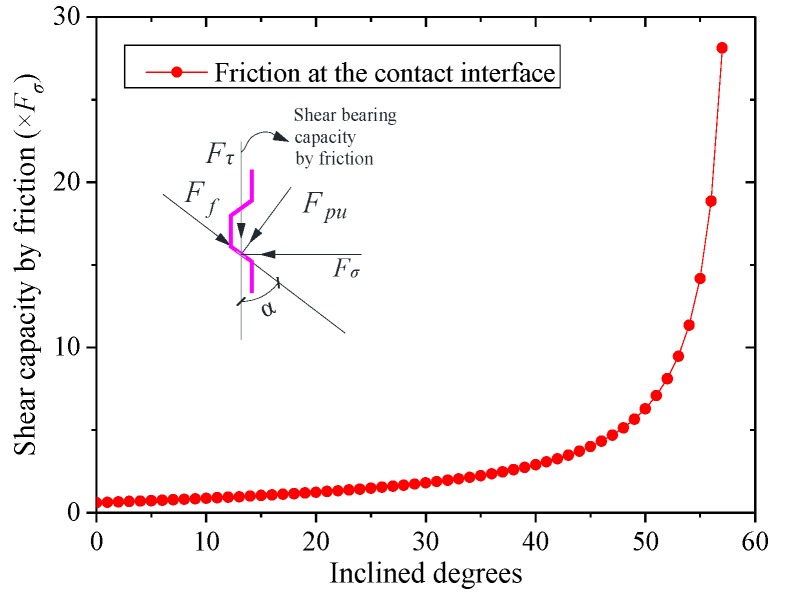
Friction varied with inclined degrees.

**Figure 14:**
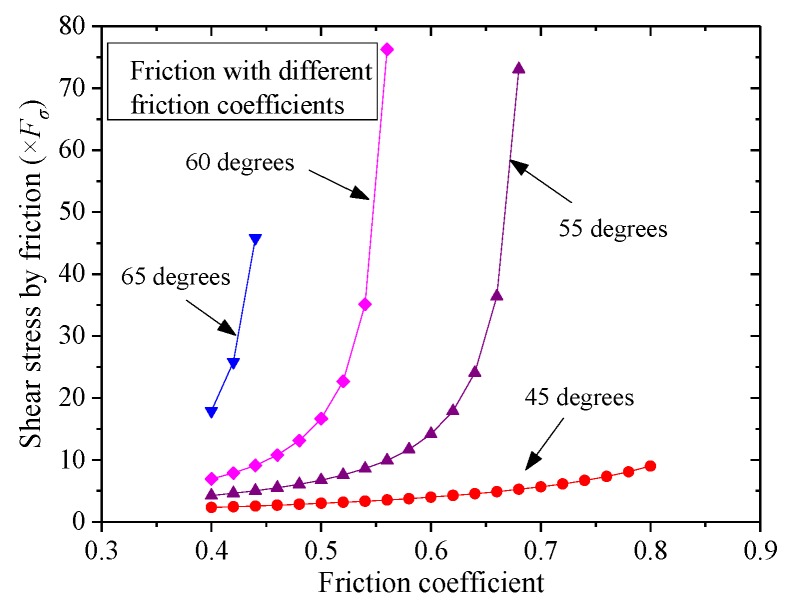
Friction with different friction coefficients.

The fracture of the shear key will be observed if the shear bearing capacity $F_{\tau }$ calculated by Equation (6) is larger than that caused by the fracture of the concrete key or the inclined angle α is so large that shear locking occurs at the interface. The friction varying with inclined degrees is shown in Figure 13, where the friction coefficient μ is taken as 0.6. The shear bearing capacity increased sharply when α was close to 60 degrees (from 50 to 60 degrees) till shear locking appeared, in which $F_{\tau }$ was infinite with the premise that there was confining stress applied to the key interface. The shear locking phenomenon without confining stress depended on μ and α. The friction coefficient μ of concrete could be taken from 0.4 to 1.1, as suggested by previous studies [3,20]. Therefore, in most cases, the shear locking appeared when α was larger than 60 degrees. In addition, the friction with different friction coefficients is shown in Figure 14. It could be found that the friction coefficient μ also significantly influenced the shear locking (μ≤tanα) and the shear bearing capacity. For the dimensions of the shear keys in this test, the shear locking may not be realized especially for that with relatively small confining stress, resulting in the slipping of the contact surface. Therefore, another reason leading to the slipping may be that this specimen had a small value of μ with a smoother surface.

### 4.2. Shear Strength of Epoxied Joints and Joints with Dowel Reinforcement 

For the joints with dowel reinforcement, due to the existence of the dowel action of inserted steel bars across the shear plane, the calculated results by the above equations were all small. TSPCS of China (2014) [8] provides the following design formulae to obtain the shear strength of horizontal joints at the bottom of a precast concrete column.

When the precast column is subjected to compression:

(8) $$V_{uE}=0.8N+1.65A_{sd}\sqrt{f_{c}f_{y}}$$

When the precast column is subjected to tension:

(9) $$V_{uE}=1.65A_{sd}\sqrt{f_{c}f_{y}\left(1-{\left(\frac{N}{A_{sd}f_{y}}\right)}^{2}\right)}$$

where $f_{c}$ is the concrete strength, $f_{y}$ is the tensile strength of inserted steel bar, N is the axial force, $A_{sd}$ is the area of inserted steel bars, and $V_{uE}$ is the shear capacity of joints under seismic design. The first term 0.8N on the right of Equation (8) presents the fiction produced by axial compression, and the second term presents the shear strength provided by the dowel action of reinforcement. The calculated results by Equation (8) are listed in Table 3. It could be found that the calculated results were close to the test results for dry flat joints. However, the calculated results of the joints with shear keys and epoxy resin were obviously smaller. Therefore, a calculated formula for computing the shear strength of dry joints $V_{DR}$ with shear keys and dowel reinforcement was proposed based on AASHTO and TSPCS.

(10) $$V_{DR}=A_{k}\sqrt{f_{c}^{\prime }}\left(0.9961+0.2048\sigma_{n}\right)+0.6A_{sm}\sigma_{n}+1.65A_{sd}\sqrt{f_{c}^{\prime }f_{y}}$$

The calculated results of the specimen M2.0-D-K2-R by Equation (10) agreed well with the test results with an error of −0.75%. The first two terms on the right of Equation (10) were mainly adopted to calculate the shear strength of keyed joints, so the calculated results were conservative for the specimens M2.0-D-F-R and M1.0-D-F-R.

For the joints with epoxy resin, it was concluded from the mentioned tests that the damaged shear surface was located at the concrete layer adjacent to the epoxy resin layer. The epoxied joints could approximately be equivalent to cast-in-place joints. Therefore, the shear strength of epoxied joints depended on the tensile strength of concrete and confining stress. For the plain concrete joints, the ultimate shear capacity would be reached once the shear crack appeared. Hence, a shear strength formula of epoxied joints was proposed according to the above equations and Figure 10b.

(11) $$V_{EP}=A_{k}\sqrt{f_{c}^{\prime }}\left(0.7282+0.1392\sigma_{n}\right)$$

When reinforcement was inserted across the key interface, the shear-friction concept was used for the calculation of the shear strength that the steel bars in tension provided clamping forces. According to ACI318-14 [7], if shear-friction reinforcement is perpendicular to the shear plane, nominal shear strength can be calculated as follows:

(12) $$V_{n}=\mu A_{vf}f_{y}$$

where $A_{vf}$ is the area of inserted reinforcement across the interface. Thus, the shear strength of joints with inserted reinforcement can be expressed as follows:

(13) $$V_{EP}=A_{k}\sqrt{f_{c}^{\prime }}\left(0.7282+0.1392\sigma_{n}\right)+\mu A_{vf}f_{y}$$

The calculated result of M2.0-E-K2-R by Equation (13) was 915 kN with an error of 11% compared to the test result (in Table 2). However, in fact, the measured shear bearing capacity of the specimen was less than the true shear capacity, as mentioned in a previous section of this article.

## 5. Conclusions

In this study, a series of tests on the shear strength of the joints with various details applied in precast shear walls were carried out with/without high strength shear reinforcement across the shear plane. The tested parameters of the specimens included the joint types, the number of shear keys, the existence of high strength steel bars inserted at the joints, and the levels of confining stress. The shear capacity, stiffness, and shear transfer mechanisms of these joints were studied. The conclusions were as follows:

1. For the specimens with a confining stress of 0.5 MPa or less than 1.0 MPa (M0.5-D-K1-N and M1.0-D-K2-N), the shear locking phenomenon did not occur in the shear key due to the adopted small lateral force, resulting in the slipping of the shear plane at the interface without shearing-off failure of the shear key. The shear strength of the shear key with the configuration could not fully be developed with small confining stress less than 1.0 MPa. The inclined angle at the end of the keys should be larger than 54 degrees at least in this study.

2. The shear strengths of joints with epoxy resin and high strength dowel reinforcement were significantly higher than those of dry joints with the same size. However, the failure mode of epoxied joints was suddenly brittle fracture after reaching the ultimate load accompanied by a vertical crack. The shear strengths of joints with inserted high strength reinforcement were greater than those of joints with only epoxy resin, and their failure modes were ductile with large deformations. 

3. A calculation method was put forward to compute the push-off shear strength for the joints with dowel reinforcement where the equations were proposed according to the equations of AASHTO and the shear-friction concept in ACI318. The calculated results agreed well with the test results.

4. When the shear bearing capacity by friction was lower than the bearing capacity of the shear key itself, slipping would be observed. The friction at the key interface was dependent on the applied confining stress $F_{\sigma }$, friction coefficient, and inclined angle α. A small confining stress and a smooth contact surface may lead to the strengths of the shear keys not being fully developed. An inclined angle α of the keys of more than 60 degrees was suggested to be designed in the interface.

## Figures and Tables

**Table 1:** Test information matrix of push-off tested specimens. M, monotonic loading; E, epoxied; K, key joint; D, dry; F flat joint; CIP, cast-in-place.

No.	Specimen	Concrete Strength $f_{c}^{\prime }$ (MPa)		Number of Keys	Confining Stress (MPa)	Dry or Epoxy Resin	Contacted State of the Interface
		Left Component	Right Component				
A1	M2.0-E-K1-N	37.8	42.2	1	2.0	Epoxy resin	Complete
A2	M0.5-E-K1-N	40.2	40.8	1	0.5	Epoxy resin	Complete
A3	M2.0-D-K1-N	38.1	40.6	1	2.0	Dry	Complete
A4	M0.5-D-K1-N	41.3	40.6	1	0.5	Dry	Partial
A5	M1.0-D-K1-N	41.3	40.6	1	1.0	Dry	Partial
B1	M2.0-E-K2-N	39.6	39.1	2	2.0	Epoxy resin	Complete
* B2	M2.0-E-K2-N	38.9	34.7	2	2.0	Epoxy resin	Complete
B3	M3.0-E-K2-N	39.2	41.7	2	3.0	Epoxy resin	Partial
B4	M1.0-D-K2-N	38.4	32.9	2	1.0	Dry	Complete
B5	M2.0-D-K2-N	40.5	40.2	2	2.0	Dry	Complete
B6	M3.0-D-K2-N	39.1	40.4	2	3.0	Dry	Complete
* C1	M2.0-E-K3-N	39.3	40.7	3	2.0	Epoxy resin	Complete
* C2	M2.0-E-K3-N	38.7	39.7	3	2.0	Epoxy resin	Complete
C3	M1.0-E-K3-N	40.3	38.4	3	1.0	Epoxy resin	Complete
C4	M2.0-D-K3-N	39.7	40.7	3	2.0	Dry	Partial
D1	M2.0-D-K2-R	39.2	38.4	2	2.0	Dry	Partial
D2	M2.0-E-K2-R	38.6	38.8	2	2.0	Epoxy resin	Complete
* E1	M2.0-E-F-R	43.5	37.5	0	2.0	Epoxy resin	Complete
E2	M1.0-E-F-R	38.8	39.9	0	1.0	Epoxy resin	Complete
E3	M2.0-D-F-R	40.5	38.5	0	2.0	Dry	Complete
E4	M1.0-D-F-R	41.5	41.8	0	1.0	Dry	Complete
CIP	M1.0-CIP-N	39	40.2	–	1.0	–	–

Note: * Specimens B2, C1, C2, and E1 were damaged by local failure at the loading end, so the ultimate shear loads of their interfaces were not obtained. The left and right components of a specimen are the female and male parts for the keyed specimens, respectively.

**Table 2:** Experimental results of the joints tested under monotonic loading.

No.	Specimen	Ultimate Shear Load (kN)	Average Shear Stress (MPa)	Average Value of Normalized Shear Stress (MPa)	Shear Strength by AASHTO (kN)	Error (%)	Rombach and Specker (kN)	Error (%)
A1	M2.0-E-K1-N	330	8.25	1.270	195.59	−40.73	157.84	−52.17
A2	M0.5-E-K1-N	260	6.50	1.018	144.29	−44.50	125.56	−51.71
A3	M2.0-D-K1-N	170	4.25	0.667	196.27	15.45	158.68	−6.66
A4	M0.5-D-K1-N	60	1.50	0.235	146.17	143.62	128.64	114.40
A5	M1.0-D-K1-N	130	3.25	0.510	165.23	27.10	141.64	8.95
B1	M2.0-E-K2-N	580	5.80	0.928	423.25	−27.03	351.76	−39.35
B2	M2.0-E-K2-N	–	–	–	420.13	–	347.84	–
B3	M3.0-E-K2-N	740	7.40	1.146	508.37	−31.30	414.52	−43.98
B4	M1.0-D-K2-N	340	3.40	0.593	331.51	−2.50	280.04	−17.64
B5	M2.0-D-K2-N	410	4.10	0.647	427.22	4.20	356.80	−12.98
B6	M3.0-D-K2-N	540	5.40	0.850	507.86	−5.95	413.96	−23.34
C1	M2.0-E-K3-N	700	7.00	1.097	572.87	−18.16	460.12	−34.27
C2	M2.0-E-K3-N	–	–	–	568.85	–	455.08	–
C3	M1.0-E-K3-N	540	5.40	0.871	478.10	−11.46	403.52	−25.27
C4	M2.0-D-K3-N	600	6.00	0.940	575.54	−4.08	463.48	−22.75
^1^ D1	M2.0-D-K2-R	638	6.38	1.030	421.47	−33.94	349.52	−45.22
^1^ D2	M2.0-E-K2-R	820	8.20	1.316	418.79	−48.93	346.16	−57.79
E1	M2.0-E-F-R	–	–	–	120.00	–	130.00	–
E2	M1.0-E-F-R	750	7.50	1.187	60.00	−92.00	65.00	−91.33
E3	M2.0-D-F-R	450	4.50	0.725	120.00	−73.33	130.00	−71.11
E4	M1.0-D-F-R	330	3.30	0.510	60.00	−81.82	65.00	−80.30
CIP	M1.0-CIP-N	550	5.50	0.867	744.53	35.37	611.00	11.09

Note: ^1^ The dowel action was not taken into account in the calculation of shear strength of Specimens D1 and D2, which only included the shear strength of plain concrete shear keys when the results were calculated by the design or calculation formulae of AASHTO and Rombach and Specker.

**Table 3:** Shear capacity of joints with dowel reinforcement.

No.	Specimen	Ultimate Shear Capacity (kN)	Calculated Shear Strength According to Equation (8) (kN)	Error (%)	Calculated Shear Strength According to Equation (10) (kN)	Error (%)	Calculated Shear Strength According to Equation (13) (kN)	Error (%)
D1	M2.0-D-K2-R	638	371.72	−41.74	633.19	−0.75	–	–
D2	M2.0-E-K2-R	820	370.09	−54.87	–	–	915	11
E1	M2.0-E-F-R	–	383.03	–	–	–	–	–
E2	M1.0-E-F-R	750	290.63	−61.25	–	–	–	–
E3	M2.0-D-F-R	450	375.20	−16.62	335.20	−25.51	–	–
E4	M1.0-D-F-R	330	297.84	−9.75	277.84	−15.81	–	–
